# Quality of life and long-term outcomes of octo- and nonagenarians following acute care surgery: a cross sectional study

**DOI:** 10.1186/1749-7922-8-23

**Published:** 2013-07-01

**Authors:** Sayf Gazala, Yvonne Tul, Adrian Wagg, Sandy L Widder, Rachel G Khadaroo

**Affiliations:** 1 Department of Surgery, University of Alberta, 2D WMC, 8440-112 St NW, Edmonton, AB, Canada T6G 2B7; 2 Department of Medicine, University of Alberta, Edmonton, Alberta, Canada

**Keywords:** Elderly, Emergency Surgery, Health Outcomes

## Abstract

**Background:**

While advanced age is often associated with poorer surgical outcomes, long-term age-related health status following acute care surgery is unknown. The objective of our study was to assess post-operative cognitive impairment, functional status, and quality of life in elderly patients who underwent emergency surgery.

**Methods:**

We identified 159 octo- and nonagenarians who underwent emergency surgery between 2008 and 2010 at a single tertiary hospital. Patients were grouped into three cohorts: 1, 2, and 3 years post-operative. We conducted a survey in 2011, with octo- and nonagenarians regarding the impact of emergency surgical procedures. Consenting participants responded to four survey questionnaires: (1) Abbreviated Mental Test Score-4, (2) Barthel Index, (3) Vulnerable Elders Survey, and (4) EuroQol-5 Dimensional Scale.

**Results:**

Of the 159 octo- and nonagenarians, 88 (55.3%) patients were alive at the time of survey conduction, and 55 (62.5%) of the surviving patients consented to participate. At 1, 2, and 3 years post-surgery, mortality rates were 38.5%, 44.7%, and 50.0%, respectively. More patients had cognitive impairments at 3 years (33.3%) than at 1 (9.5%) and 2 years (9.1%) post-operatively. No statistical difference in the ability to carry out activities of daily living or functional decline with increasing time post-operatively. However, patients perceived a significant health decline with the greater time that passed following surgery.

**Conclusions:**

Our study showed that half of the patients over the age of 80 are surviving up to 3 years post-operatively. While post-operative functional status appears to be stable across the 3 cohorts of patients, perceived health status declines over time. Understanding the long-term post-operative impact on cognitive impairment, functional status, and quality of life in elderly patients who undergo acute care surgery allows health care professionals to predict their patients’ likely post-operative needs.

## Background

By 2040, 1 in 4 American and Canadians will be over age 65, nearly double today’s senior population, with about 19 millions over the age of 85 years in the United States [1-3]. In the developed regions of the world life expectancy is projected to increase and reach on average about 80 years [4]. These older patients are presenting for surgical evaluation of acute illness in increasing numbers [5]. Acute diseases requiring emergency surgical intervention are more risky than elective procedures given individuals’ age, comorbidities, as well as their acute physiological changes [6]. Many of these elderly patients therefore present unique medical challenges, often with a significant burden of pre-existing illness, poly-pharmacy, frailty, as well as limited social support. Acute surgical services, designed to address acute problems with rapid diagnosis and turnover, may fail older people who require longer-term support, restorative care and follow-up, even from so-called “minor” surgical procedures.

Assessment of function and frailty in the elderly is gaining popularity as a predictor of outcomes in older patients undergoing surgery [7,8]. Functional capacity indicates a person’s ability to carry out everyday tasks [9]. It provides a measure of independence, which is of particular concern to seniors’ health related quality of life (HRQOL). Functional capacity takes into account both basic activities of daily living (ADLs) – eating, bathing, dressing, toileting, walking – and instrumental activities of daily living (IADLs) – shopping, banking, housekeeping [10]. Unfortunately, it is not always possible to perform a comprehensive pre-surgical assessment in the emergency setting. Frail elderly patients are often associated with poorer surgical outcomes and increased morbidity (surgical site infections, end organ dysfunction, anastomosis leakage, and sepsis), post-operative delirium and in-hospital falls [11,12], however long term age-related health status following acute care surgery (ACS) is unknown. To date there has been limited published reports of post-operative outcomes following ACS in older patients. We conducted a cross sectional study in an older cohort to provide quantitative data regarding the long-term impact of emergency procedures. We wanted to assess the presence of cognitive impairment; functional status, frailty and health related quality of life in elderly patients who underwent ACS.

## Methods

We retrospectively identified 159 octo- and nonagenarians who underwent emergency surgeries between 2008 and 2010 under a specialized emergency service at a single tertiary center (University of Alberta Hospital’s Acute Care Emergency Surgery (ACES) service, Edmonton, Alberta). The service is unique in that there is a fully functional theatre and team dedicated to emergency general surgery cases exclusively during day time hours, in addition to the emergency after hours. Older patients (≥65) comprise a significant proportion of those admitted to our ACES service with up to one third of these patients being greater than the age of 80 and account for 25% of annual operations. Typical surgical procedures include: colonic surgery- resection and diversion (23%), small bowel surgery- adhesions and resection (20%), exploratory and trauma laparotomy (16%). cholecystectomy (10%), Hernia surgery- incarceration and strangulation (9%), duodenal ulcer surgery- bleeding and perforation (5%), and other less commonly performed procedures (17%)

A cross sectional study design was implemented based on the year the surgical procedure was performed. Patients were then grouped into three groups based on the duration since the surgery: *Group 1* included patients who had an emergency procedure in 2010 and were contacted 1 year post-op; *Group 2* included patients who had an emergency procedure in 2009 and were contacted 2 years post-op; *Group 3* included patients who had an emergency procedure in 2008 and were contacted 3 years post-op.

After identifying those patients who are still alive, the three cohorts of patients were contacted by telephone to conduct the survey (up to three attempts). Follow-up calls were completed between November 2011 and January 2012. Participants who were hard-of-hearing were mailed surveys and asked to return them in pre-paid envelopes. In some instances, a surrogate (spouse or relative) was used if the patient was unable to respond (demented or no English).

Consented participants, or their surrogates, responded to the following four survey questionnaires: (1) Abbreviated Mental Test Score-4 (AMTS-4), a brief 4-item survey that screens for cognitive impairment. Patients are considered cognitively impaired if they fail to answer any of the four questions [13]. (2) Barthel Index, a 10-item questionnaire with three levels of answers, which assesses the level of independence with activities of daily living [14,15]. (3) Vulnerable Elders Survey (VES-13), is a 13-item questionnaire that measures frailty in older persons. It has a maximum score of 10 (high score indicates worse health state). The VES-13 has been validated in elderly patients to predict death and decline in function [6,16,17]. (4) EuroQol-5 Dimensional Scale (EQ-5D), a health utility measure that has five questions with three levels of answers for each question, and can yield a health state between 0 and 1 (where 0 is death and 1 is the best health state a person can have). The EQ-5D is a valid and reliable tool for the measurement of health related quality of life [18]. The four questionnaires have been used and are reliable in this patient population group. The results will give a clear indication on cognitive function, independence, activity of daily living, and health related quality of life in general. In addition, participants were asked whether they currently live alone, and whether their place of residence had changed since the time of their surgery.

The study received ethical committee approval from the HREB at the University of Alberta. STATA data analysis and statistical software, version 12, was used for the statistical analysis. ANOVA was used to assess the difference between the three groups for each of the questionnaires. A p value of <0.05 was used to assign statistical significance for comparisons.

## Results

A total of 159 octo- and nonagenarians were operated on under the ACES service during the study period (approximately 7% of the total volume). 88 (55.3%) patients were alive at the time of follow-up. For those patients contacted at 1 year following surgery (group 1) (N=52), there was a 38.5% mortality rate. At 2 years post-surgery, group 2, (N=47), there was a 44.7% mortality rate, and at 3 years post-surgery, group 3, (N=60), there was a 50.0% mortality rate. Fifty-seven (64.8%) of the surviving patients consented to participate in the follow-up survey, 23 (71.9%) from Group 1, and 16 (61.3%) from Group 2 and 16 (53.3%) from Group 3 (Table 1). Fifteen were excluded because of dementia and/or institutionalization, refusal to participate, or an inability to speak English and lack of access to an interpreter. Seven were lost to follow up.

**Table 1:** The three cohorts included in the analysis

	**No. death (%)**	**No. alive (%)**	**No. included (%)**	**No. excluded (%)**	**Reasons for exclusion**
Group 1	20 (38.5)	32 (61.5)	23 (71.9%)	9 (28.1)	-Loss to follow up
					-Dementia
					-Refusal
Group 2	21 (44.7)	26 (55.3)	16 (61.5)	10 (38.5)	-Loss to follow up
					-Dementia
					-Refusal
Group 3	30 (50)	30 (50)	16 (53.3)	14 (46.7)	-Loss to follow up
					-Dementia
					-No English
					-Refusal

### Demographics and geographical location

In Group 1, there were 7 females (mean age 83.4, SD 1.7) and 9 males (mean age 81.3, SD 1.2). More than half of the respondents (60.9%) were living with someone, usually a spouse or a family member. In Group 2, there were 8 females (mean age 83.1, SD 2.6) and 8 males (mean age 83.2, SD 3.1). Less than half of the respondents (43.8%) were living with someone. In Group 3, there were 13 females (mean age 83.4, SD 2.7) and 10 males (mean age 83.4, SD 2.3). Half of them were living with someone. Demographic characteristics of the groups are shown in Table 2.

**Table 2:** Demographic characteristics of the three groups

	**Sex (M:F)**	**Age (mean, (SD))**	**Living alone (%)**
Group 1	Male 9	81.3 (1.2)	(60.9)
	Female 7	83.4 (1.7)	
Group 2	Male 8	83.2 (3.1)	(43.8)
	Female 8	83.1 (2.6)	
Group 3	Male 10	83.4 (2.3)	(50.0)
	Female 13	83.4 (2.7)	

### Cognitive status

**Figure 1:**
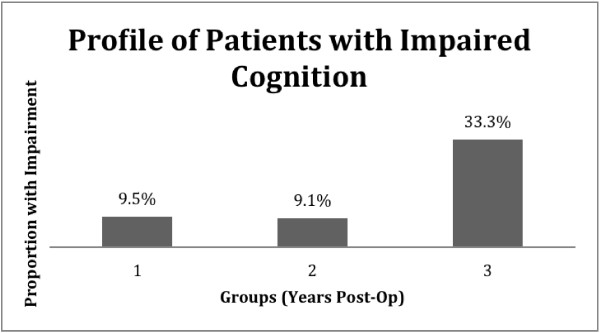
**Using the AMTS-4, a score between 0–3 indicates impaired cognition; a score of 4 indicates normal cognition.**

Data from the abbreviated mental test score-4 (AMTS-4) indicate that more patients had cognitive impairments at 3 years (33.3%) than at 1 (9.5%) and 2 years (9.1%) following ACS (See Figure 1). There is a statistically significant difference between the proportion of those with cognitive impairment at 3 years post-operatively and that at 1 and 2 years after surgery (p value =0.05). We found no statistically significant difference comparing the proportion of men and women with cognitive impairment combining the three groups, Odds Ratio of 1.3 (p = 0.18).

### Functional status

There was no statistical difference among the three groups in their ability to carry out activities of daily living or functional decline post-operatively. There were no statistically significant differences in functional status between females and males comparing either the Barthel Index score or the VES-13 over the three years following ACS.

### Health related quality of life

**Figure 2:**
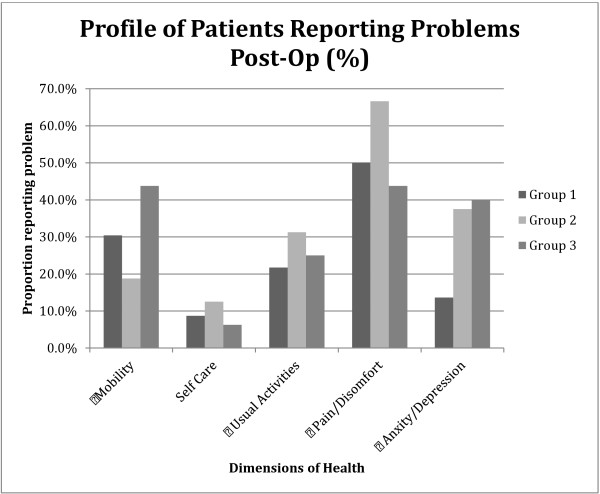
**Quality of Life as measured by the EQ**-**5D questionnaire for the three groups.**

**Figure 3:**
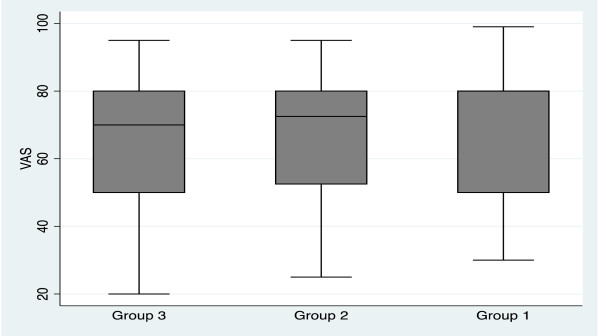
**EQ-5D Visual Analogue Scale comparing the three cohorts.**

The mean (SD) EQ-5D for the three groups was 0.83 (0.2) for Group 1, 0.77 (0.17) for Group 2, and 0.82 (0.2) for Group 3. There was no statistical significance between scores in the three groups. Figure 2 describes the patients who reported problems with each of the five dimensions of the EQ-5D (moderate and severe problems). Perceived health state of the patients from the visual analogue scale (VAS) of the EQ-5D, was higher in patients who had more recent surgery (Group 1) than the other groups, but the difference was not statistically significant (See Figure 3).

## Discussion

Investigating ways to optimize health care for elders is important to maximize quality of life and reduce the burden of comorbid disease, functional and cognitive impairment on society. In the next 35 years, 1 in 4 North Americans and Europeans will be over the age of 65 years. These changing demographics need to alter the way we think about and how we deliver healthcare. There are an increasing proportion of elderly patients presenting to our acute care hospitals who often also have multiple comorbidities; unfortunately most current models of health care delivery do not take into account the aforementioned. In order to provide health care specific to the elderly, accurate data on outcomes from acute emergency surgical interventions is needed. There has to date been limited attempts to measure change in the quality of life of the elderly following surgery and few reports that considers return to home and normal function following acute surgical intervention [19,20]. These factors are probably the most important to consider in this group. How early patients return home, their level of physical and cognitive function, the amount of support they need and their discharge destination are of critical importance to healthcare planners who need to allocate resources in a political and social environment where expectations are high and where costs and resource limitations need to be taken into consideration.

Results from our mid-term follow-up revealed that greater than half of patients greater than 80 years who underwent emergency surgery survived up to 3 years post-operatively. Post-operative functional status appeared to be stable across the 3 cohorts of patients, regardless of time of assessment post surgery. This evidence supports that good surgical outcomes is possible even in the elderly, and that much more must be considered than simply age alone. Although there was a cognitive decline at 3 years post-operatively compared to 1 and 2 years following surgery, this difference was not statistically significant.

Overall, there was moderate variability in the reported limitations in functional capacity of our sample of elderly patients, underlining the diversity of this acute care population. With age, losses in functional capacity become more common and are increasingly severe. Most people with a limitation in functional capacity, when younger than 85 years, report only mild limitations. However, 25% of seniors 85 years and over report a moderate (15%), severe (5%), or total (5%) limitation in functional capacity [1]. Our sample reported no decline in their HRQOL following surgery but also had a significantly better HRQOL compared to the general elderly population of Alberta (greater than75 years), this most likely can be explained by multiple factors. One of the most important being, patients with better HRQOL are more likely to undergo an emergency surgical intervention when compared to those with lower HRQOL at baseline. Additionally, patients with better HRQOL are more likely to respond to our study surveys.

There are several limitations to this study including the retrospective nature of the study that will limit the data available for analysis, the presence of selection and survivor biases. As well, we specifically only examined the outcomes of those elderly patients who had a surgical intervention. We did not include those patients with acute surgical conditions who were treated conservatively. Other factors such as socioeconomic status, type of residence (rural vs. urban), and professional background might have a confounding effect on the results of this analysis and were not accounted for in this analysis.

Our study also was not designed to measure pre- to post-acute care changes in cognitive impairment, functional status, or quality of life. Rather, the intent was to get a “snapshot” of how elderly patients fare after surgery and assess the feasibility of collecting data from this elderly, more vulnerable group. For this reason, it is not possible to assess what impact ACS might have had on our patients’ level of independence and quality of life. We are currently undertaking a prospective study, which addresses these limitations in order to provide greater insight on the effects of ACS on this elderly population.

## Conclusion

Our research demonstrates that acute care surgery patients over 80 years of age had a greater than fifty percent survival rate at 3 years post-operatively, and of those elderly patients who survived had a stable health related quality of life and functional status. Understanding the characteristics of the geriatric acute care surgery population allow health care professionals to deliver more effective services to older patients.

## Competing interests

The authors declare that they have no competing interests.

## Authors’ contributions

SG: Study design, data analysis, data interpretation, and writing, YT: data collection, writing, AW: study design, data interpretation, and critical revision, SLW: Study design, data interpretation, and critical revision, RGK: Study design, data analysis, data interpretation, and critical revision. All authors read and approved the final manuscript.
